# Transplantation of Embryonic Neural Stem Cells and Differentiated Cells in a Controlled Cortical Impact (CCI) Model of Adult Mouse Somatosensory Cortex

**DOI:** 10.3389/fneur.2018.00895

**Published:** 2018-10-24

**Authors:** Mohammad Nasser, Nissrine Ballout, Sarah Mantash, Fabienne Bejjani, Farah Najdi, Naify Ramadan, Jihane Soueid, Kazem Zibara, Firas Kobeissy

**Affiliations:** ^1^Biology Department, Faculty of Sciences-I, Lebanese University, Beirut, Lebanon; ^2^ER045, PRASE, DSST, Lebanese University, Beirut, Lebanon; ^3^Department of Biochemistry and Molecular Genetics, Faculty of Medicine, American University of Beirut, Beirut, Lebanon

**Keywords:** TBI, NSC, neurosphere, astrocyte, neuron, lesion, transplantation, cortex

## Abstract

Traumatic brain injury (TBI) is a major cause of death worldwide. Depending on the severity of the injury, TBI can reflect a broad range of consequences such as speech impairment, memory disturbances, and premature death. In this study, embryonic neural stem cells (ENSC) were isolated from E14 mouse embryos and cultured to produce neurospheres which were induced to generate differentiated cells (DC). As a cell replacement treatment option, we aimed to transplant ENSC or DC into the adult injured C57BL/6 mouse cortex controlled cortical impact (CCI) model, 7 days post-trauma, in comparison to saline injection (control). The effect of grafted cells on neuroinflammation and neurogenesis was investigated at 1 and 4 weeks post-transplantation. Results showed that microglia were activated following mild CCI, but not enhanced after engraftment of ENSC or DC. Indeed, ipsilateral lesioned somatosensory area expressed high levels of Iba-1+ microglia within the different groups after 1 and 4 weeks. On the other hand, treatment with ENSC or DC demonstrated a significant reduction in astrogliosis. The levels of GFAP expressing astrocytes started decreasing early (1 week) in the ENSC group and then were similarly low at 4 weeks in both ENSC and DC. Moreover, neurogenesis was significantly enhanced in ENSC and DC groups. Indeed, a significant increase in the number of DCX expressing progenitor cells was observed at 1 week in the ENSC group, and in DC and ENSC groups at 4 weeks. Furthermore, the number of mature neuronal cells (NeuN+) significantly increased in DC group at 4 weeks whereas they decreased in ENSC group at 1 week. Therefore, injection of ENSC or DC post-CCI caused decreased astrogliosis and suggested an increased neurogenesis via inducing neural progenitor proliferation and expression rather than neuronal maturation. Thus, ENSC may play a role in replacing lost cells and brain repair following TBI by improving neurogenesis and reducing neuroinflammation, reflecting an optimal environment for transplanted and newly born cells.

## Introduction

The complexity of the cerebral cortex in terms of cell specificity and projections is revealed by the difficulty to repair damaged areas caused by injuries or diseases within the central nervous system (CNS). Indeed, the loss of cortical neurons is a common characteristic to many neuropathological conditions such as lesions (trauma, stroke) or neurodegenerative diseases (amyotrophic lateral sclerosis, Huntington's disease) and is associated with irreversible functional deficits and cell death (1, 2). Traumatic brain injury (TBI) is a worldwide silent epidemic problem leading to the disability and sometimes the death of patients (3). Annually, one out of 200 people suffers from TBI (4) and an estimated 1.5 million people lose their lives due to TBI (5). Mild TBI, which accounts for ~75% of all head injuries (6), is a concussion that causes neurobiological modifications in the CNS (7), short-term confusion, cognitive impairments, and transient unconsciousness (8). According to pathology, TBI causes primary injury due to mechanical forces at the time of the incident followed by a secondary injury. The latter is characterized by cytotoxicity, cerebral edema, oxidative stress, apoptosis, mitochondrial dysfunction, and inflammation (9–11). After brain injury, the blood-brain barrier (BBB) is disrupted allowing the migration of immune cells to the site of injury associated with long-lasting neuronal dysfunction and cell death (12).

Many studies have shown spontaneous functional recovery after TBI (13); however, it remains limited (14). Animal models mimicking patients TBI have been established to better understand TBI pathophysiology and to develop potential treatments (15–19). Several preclinical studies have targeted secondary injury events in animal TBI models to test the therapeutic efficacy of drugs such as calcium channel blockers, inhibitors of lipid peroxidation, excitatory amino acid inhibitors and others (20). Except for few limited treatments relieving specific symptoms, especially in acute animal models, available TBI therapies were either ineffective on patients or didn't reach clinical trials (21–24). The role of stem cells in tissue reconstruction, neuroprotection and trophic support to the host tissue makes them a potential therapeutic option (25). Recently, neural transplantation as a prospective therapy for TBI has emerged using mesenchymal stem cells (MSCs) (26), neural stem cells (NSCs) (27–30), induced pluripotent stem cells (iPSCs) (31) and embryonic neural stem cells (ENSCs) (32).

In response to TBI, the capacities of axonal regrowth and spontaneous regeneration of the CNS becomes limited. ENSCs are the optimal cell source for neural transplantation owing to their unlimited capacity of self-renewal and plasticity. When transplanted into damaged brain areas, these cells are capable of differentiation, migration, and innervation (32). A recent study confirmed that transplantation of pre-differentiated human ENSCs increased angiogenesis and reduced astrogliosis along with improving long-term motor function (33, 34). Another study established that transplantation of differentiated human ENSCs into severely injured controlled cortical impact (CCI) rat brains resulted in neuronal survival at the lesioned area, reduction in astrogliosis and enhancement in angiogenesis (33). Moreover, it was shown that transplantation of embryonic cortical neurons, immediately after injury in the adult motor cortex, allows the anatomical reconstruction of injured motor pathways and the development of projections to appropriate cortical and subcortical host targets (35, 36). Furthermore, it was demonstrated that cortical progenitors are capable to develop into specific neuronal phenotypes and to express specific transcription factors according to their developmental program (37). Neuronal transplantation appears as a promising therapeutic strategy to replace neurons and damaged pathways. Its effectiveness depends on the capacity of grafted cells to develop appropriate cellular populations and to integrate into host preexisting neuronal circuitry. Understanding the spatiotemporal development of different cell populations within the graft, and their axonal outgrowth, is of crucial importance.

In fact, TBI can lead to both sensorimotor and cognitive deficits (38). It could be hypothesized that many of the deficits observed after TBI, whether cognitive or sensory, are aggravated by damages in sensory processing (39). Multiple studies used different models of trauma or ischemia in the somatosensory cortex and aimed to determine the reproducibility of these models for behavioral and functional consequences after the injury (40, 41). In the present study, we aimed to establish a mCCI model targeting the primary somatosensory cortical area followed by studying the consequences of transplantation of embryonic neural stem cells (ENSCs) or differentiated cells (DCs) on inflammation and neurogenesis.

In this study, following mCCI, we hypothesized that cell therapy by transplantation of ENSCs or differentiated cells (DCs) can reduce neuroinflammation and promote neurogenesis in the adult injured cortex. Therefore, a mCCI was performed in the cortical somatosensory area of adult C57BL/6 mice followed by transplantation, 7 days post-injury, with either an injection of ENSCs, DCs, or saline. Using specific markers, the capacity of transplanted cells to improve injured cortical area, replace lost cells and decrease neuroinflammation was then investigated after CCI. Furthermore, a time-course analysis was performed at 1 and 4 weeks post-transplantation comparing the response to the transplantation of ENSCs and DCs after mCCI.

## Methods

### Animals

This study was approved by the Institutional Animal Care and Utilization Committee (IACUC) of the American University of Beirut (AUB). All animal experimental procedures and housing were carried out in accordance with the guidelines of the Agriculture Ministry and the European Communities Council Directive (2010/63/EU). All experiments were conducted in compliance with current Good Clinical Practice standards and in accordance with relevant guidelines and regulations and the principles set forth under the Declaration of Helsinki (42). All efforts were made to reduce the number of animals used and their suffering. Female mice were coupled with males for one night before removal of the male. Females were then tested for vaginal plug and positive mice were considered pregnant. A total of 56 mice were used in this study: 8 C57BL/6 pregnant mice at E14 were used as donors of ENSCs and DCs, 16 mice were used as controls (TBI/injected with saline) and divided into 2 time points (1 and 4 weeks, *n* = 8 each), 16 mice were transplanted with ENSCs after TBI and divided into 2 time points (1 and 4 weeks, *n* = 8 each), and 16 mice were transplanted with DCs after TBI and divided into 2 time points (1 and 4 weeks, *n* = 8 each).

### Establishment of the experimental CCI *in-vivo* model

A CCI device (Leica Angle Two System, Leica microsystem, USA) was used to produce TBI in mice as described previously (43). Briefly, adult (6–8 weeks old) C57BL/6 mice (~20 g, *n* = 48, Jaxon laboratories, Maine, USA) were anesthetized with a mixture of xylazine (90 mg/kg, Panpharma) and ketamine (10 mg/kg, Interchemie), injected intra-peritoneally. The animal was held in the instrument U frame with the head at the closed end of the “U.” Animals were supported in a stereotactic frame in a prone position and secured by ear and incisor bars. To secure the animal during surgery, the ear bars, nose clamp, and incisor bar were attached to the U frame. Thereafter, a midline scalp incision was made and the skin was retracted from the skull surface. Using the software associated with the device, a target site between bregma and lambda was set, where craniotomy was made. A unilateral (ipsilateral to the site of impact) craniotomy (2 mm diameter) was performed adjacent to the central suture, midway between bregma and lambda (ML: 1.88 mm, AP: −0.57 mm, DV: −1.58 mm). To produce mild CCI in the somatosensory area, an impact was induced by an impactor of 1 mm diameter with an impact velocity of 4 m/sec, a depth of 1 mm and a dwell time of 150 ms, after which the surgical site was sutured. CCI was always performed at 7 days prior to cell injection. ENSCs were injected after 14 days of cell culture (P2) whereas DCs were injected 21 ± 3 days thereafter.

### Experimental design

A total of 48 mice, divided into 6 groups of 8 mice each, were used in this study. After 7 days of CCI lesion establishment, two groups of 8 mice each received a single injection of either saline, embryonic stem cells (ENSC, 1.5 × 10^5^ cells) or differentiated cells (DCs, 1.5 × 10^5^ cells). Injection of cells or saline was performed using a Hamilton syringe in a total volume of 3 μl. One group of mice was sacrificed after 7 days of injection (1 week) while the second group was left for 28 days (4 weeks). A total of 4 mice from each group and time point were used for immunofluorescence (IF). Ipsilateral and contralateral cortical or hippocampal brain tissue samples were then harvested and kept at −80°C for further analysis.

### Harvesting ENSCs from E14 embryos

ENSCs were isolated from E14 embryos after anesthesia of pregnant females. Briefly, heads were separated from the embryos at the level of cervical spinal cord, a horizontal cut made at the level of the eyes and then midline from the forehead toward the back of the head. Brains were removed by pushing the edges of the cut section, held steady and then a cut was performed through the cortex of each hemisphere, originating from the olfactory bulbs to the back of the hemisphere. Ganglionic eminences (GE) containing the ENSCs were then exposed and micro-dissected.

### Neurosphere assay

The harvested GE tissue is dissociated by gentle pipetting in 3 ml complete media (F12-DMEM, Sigma) supplemented with 3% B27 (50X), 0.5% N2 (100X), 1% ABAM, 1% glutamax and 1% BSA (all from Gibco). The mixture was then centrifuged for 5 min at 110 g and the pellet was resuspended in 1 ml complete media. After counting the cells using a hematocytometer, a total of 200,000 cells were seeded in T25 flasks containing 5 ml of complete media supplemented with 10 μl EGF (20 ng/ml) and incubated at 37°C and 5% CO_2_. At this stage, the obtained cells (Passage 0/Day 0, P0/D0) would grow into primary neurospheres, which takes 6–7 days to observe in the flask. After 3 days (D3) of culture, 1–2 ml complete media supplemented with 2 μL EGF (20 ng/ml) were added. At D 6–7, neurospheres were observed and were ready for the first passage. The suspensions of neurospheres were then collected, centrifuged at 110 g for 5 min. A total of 1 ml trypsin-EDTA (0.05%) was then added on the neurosphere pellet for about 2–3 min at 37°C and then its activity was stopped using 2 ml DMEM. The suspension of dissociated cells was then centrifuged at 110 g for 5 min, re-suspended in 1 ml complete media, and counted using a hematocytometer. A total of 4 × 10^5^ cells were then distributed in T25 flasks containing 5 ml of complete media supplemented with 10 μl EGF (20 ng/ml), and incubated at 37°C and 5% CO_2_. At this stage, the obtained cells (P1/D7) would grow into secondary neurospheres. This time point (D7) is the day of performing CCI. Three days after the first passage (D10), 1–2 ml complete media supplemented with 2 μL EGF (20 ng/ml) were added to each flask. Seven days after their passage (D14), secondary neurospheres were ready for injection.

### Neuroblast assay (NBA)

To produce differentiated cells (DCs), the neuroblast assay was used which is divided into 2 parts: the proliferation stage and the differentiation stage. During the proliferation stage (3–4 days), cells obtained from secondary neurospheres (P2) were plated at a concentration of 4 × 10^5^ cells/ml in the above ENSC complete media supplemented with 20 ng/ml EGF and 5% heat-inactivated FCS (Sigma). Culture flasks were then incubated in a 37°C humidified incubator with 5% CO_2_. The obtained cells proliferated and grew in a monolayer attached to the substrate. During the differentiation stage (3–4 days), when cells became 90% confluent, the media of each flask was switched to ENSC complete media supplemented with 5% FCS, without any growth factors. Culture flasks were again incubated in a 37°C humidified incubator with 5% CO_2_. Neuronal progenitors appeared on top of an astrocytic monolayer. These neuronal progenitors proliferated and produced colonies of immature neuronal cells.

### Immunocytochemistry (ICC) of neurospheres

Secondary neurospheres were fixed with 4% PFA for 20 min and then washed twice in PBS (1X) for 5 min. Cells were then resuspended and washed in PBST (0.5% Triton-X in PBS 1X) 3 times for 10 min each. Thereafter, neurospheres were blocked (3% BSA+ 10% HS in PBST) for 20 min at room temperature and then rinsed again with PBST 3 times for 10 min each. Cells were then incubated for 2 h with primary antibodies against glial fibrillary acidic protein GFAP (rabbit anti-mouse polyclonal antibody PAb, Abcam, 1/1,000) and Nestin (goat anti-mouse polyclonal antibody, Abcam, 1/3,000), washed 3 times in PBST for 10 min each and incubated with appropriate secondary antibodies (goat anti-rabbit, Cruz Fluor 594, 1/1,000 and rabbit anti-goat, Alexa Fluor 488, Abcam, 1/500) for 1 h at room temperature. The nuclei were counter stained with Hoechst for 10 min and washed 3 times in PBST for 10 min each. Finally, stained neurospheres were mounted on microscopic slides and revealed by a fluorescent microscope (Axio Observer Inverted Microscope, Carl Zeiss, Germany).

### Immunocytochemistry (ICC) of differentiated cells (DCs)

Cells in NBA cultures were validated by a double immunostaining assay using glial and neuronal specific markers. Cells were first fixed with 4% PFA for 25 min and then washed twice in PBS (1X) for 5 min. DCs were permeabilized in PBST (0.1% Triton-X in PBS 1X) 3 times for 10 min each then blocked (5% FBS in PBST 1X) for 30 min at room temperature. DCs were then rinsed with PBST 3 times for 10 min each, followed by subsequent midnight incubation with primary antibodies against GFAP (mouse anti-GFAP, Abcam, 1/1,000), Tuj1 (rabbit anti-mouse PAb to beta-Tubulin III, Abcam, 1/2,000) and Neuronal Nuclei NeuN (rabbit anti-mouse PAb to NeuN, Abcam, 1/1,000). Appropriate secondary antibodies (donkey anti-mouse PAb, Alexa-fluor 488, 1/1,000 and goat anti-rabbit, Alexa Fluor 488, 1/1,000, Abcam) were added for 1h at RT followed by washing the cells in PBST (3x, 10 min each). Hoechst was then added for 5 min to counter stain the DCs nuclei which were then washed in PBST (2x for 10 min each). Finally, DCs were mounted on microscopic slides and revealed by a fluorescent microscope (Axio Observer Inverted Microscope, Carl Zeiss, Germany).

### Transplantation

**Figure 1:**
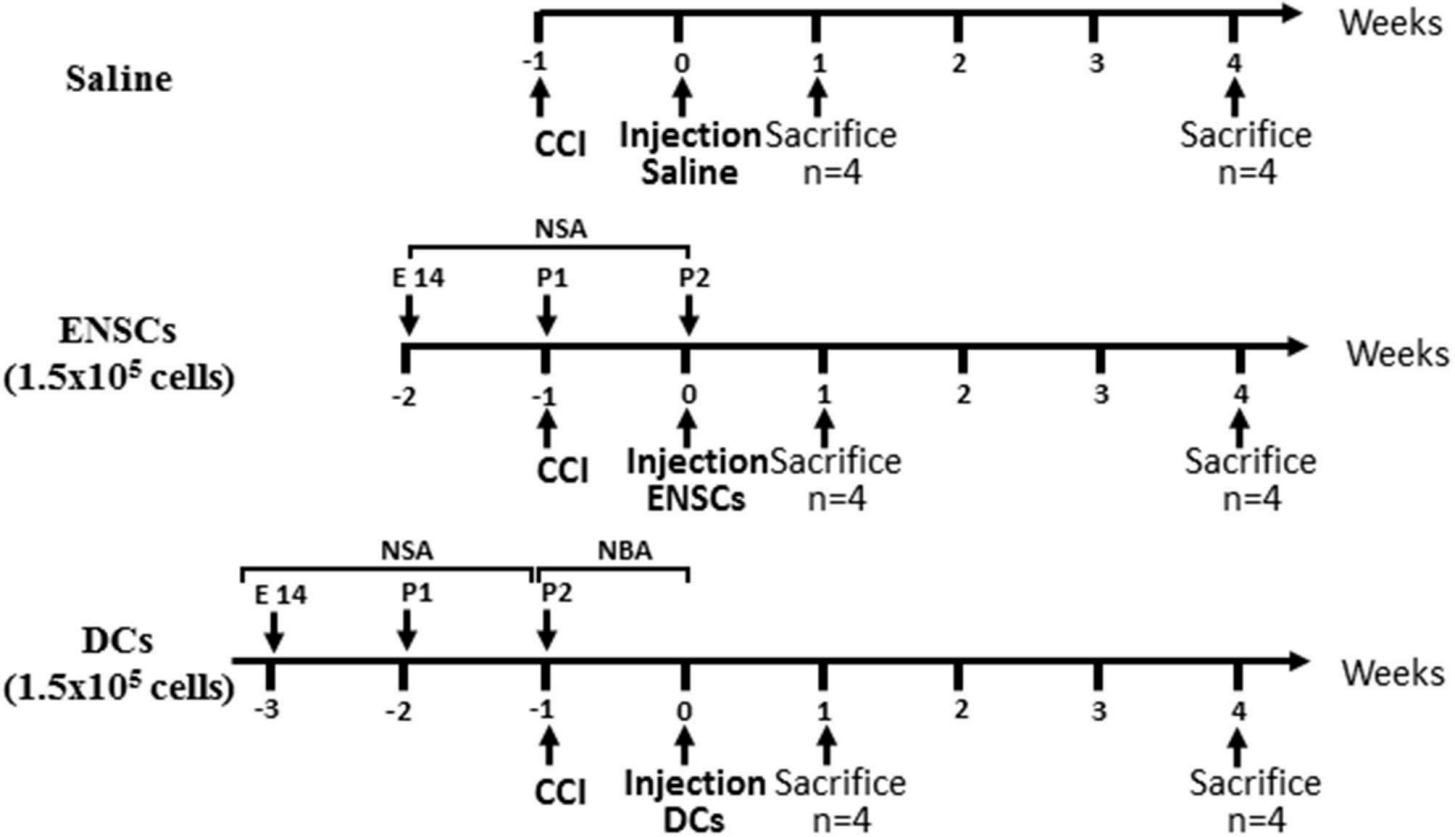
Flow Diagram of the experimental design. Mice were divided into 3 groups injected with either saline or 1.5 × 10^5^ ENSCs or DCs. Each group of mice was then divided into 2 time points, sacrificed after either 1 or 4 weeks of injection (*n* = 4 each).

After cell culture, ENSCs and DCs were ready to be injected in mouse brains at the somatosensory area injury site. After cell counting, a total of 1.5 × 10^5^ cells were prepared in 3 μL DMEM in a Hamilton syringe for injection into each mouse. Adult (6–8 weeks old) C57BL/6 mice (*n* = 48, Jaxon laboratories, Maine, USA) were used as recipients after establishing TBI lesion. The origin of cortical donor cells was from embryonic day 14 C57BL/6 mice (*n* = 8) embryos. Briefly, the mouse was anesthetized, placed in the stereotaxic frame and the CCI machine impactor was replaced with the Hamilton syringe containing the 3 μL suspension of cells. A midline incision was made to expose the skull, then Bregma and Lambda points were located manually using the Hamilton syringe. The latter is then moved to “zero” in the instruments mediolateral (ML) and anteroposterior (AP) coordinates; at this point, it is right above the target site. Using the dorsoventral (DV) drive, the syringe is lowered until it reaches the lesion site (coordinates; ML: 1.88 mm, AP: −0.57 mm, DV: −1.58 mm). This is followed by lowering the DV axis by 1 mm to perform the injection at the lesion core. Following the establishment of CCI lesion, mice were maintained for 1 week and then divided into 3 groups (*n* = 8 each) which received a single injection of either saline, ENSCs or DCs (at 1.5 × 10^5^ cells). Each group of mice was then divided into 2 time points (*n* = 4 each), sacrificed after either 1 or 4 weeks of injection (Figure 1).

### Perfusion and brain tissue preparation

Control mice without transplantation (CCI), and mice at different time points after transplantation of either ENSCs (CCI+ENSC) or DCs (CCI+DC) (day 7 and day 28) were anesthetized and their brains perfused, removed and sectioned into brain slices. Briefly, mice were injected intra-peritoneally with a mixture of xylazine (90 mg/kg, Panpharma) and ketamine (10 mg/kg, Interchemie), and perfused transcardially with 20 ml PBS (1X) followed by 30 ml of ice-cold paraformaldehyde (PFA, 4%). Brains were then removed and fixed in 4% PFA overnight. They were cut into 40 μm coronal sections with a microtome (Leica Microsystems, USA) and stored at 4°C in 0.01% sodium azide (97.5 mg of NaN3 in 100 mL PBS 1X). Brain sections were used to perform histological observations such as immunofluorescence (IF), Cresyl violet (Nissl stain) and Haematoxylin and Eosin staining (H&E).

### Immunofluorescence

This was performed as previously described (44). Briefly, brain slices were first washed twice with PBST (0.1% Triton in PBS, 1X) for 5 min each, at room temperature (RT) and on a shaker. A blocking solution (5% FBS in PBST) was then added (1 mL/well) for 1h with continuous shaking before adding the primary antibody (500 μL/well), which was left overnight at 4°C. Polyclonal antibodies used were: rabbit anti-mouse GFAP (1:1,000, Abcam), goat anti-rabbit doublecortin (DCX, 1:500, Santa Cruz), a microtubule-associated protein localized in somata and processes of migrating and differentiating neurons, rabbit anti-mouse ionized Ca binding adaptor molecule (Iba-1, 1/1,000, Abcam) and rabbit anti-mouse NeuN (1/1,000, Abcam). After incubation, sections were washed for 3 times with PBST (15 min each, RT, on a shaker), and then incubated for 1h at RT in the appropriate secondary antibody (500 μL), previously diluted in blocking solution. The secondary antibodies used were either goat anti-rabbit PAb (Alexa-Fluor 488, 1/1,000, Abcam) or rabbit anti-goat PAb (Alexa Fluor 488, 1/500, Abcam) specific for primary antibodies. Hoechst stain was then added (1 ml) for 5 min and sections were washed twice for 5 min each at RT and on a shaker. Slices were then mounted on microscope slides (star frost) using a mounting solution (2–3 drops) and a cover slip. Slides were then evaluated and photographed using a fluorescent microscope (Axio Observer Inverted Microscope, Carl Zeiss, Germany). Appropriate negative controls were performed in each assay.

### Hematoxylin and eosin (H&E) stain

Brain tissues were transferred into coated slides (star frost) and organized from anterior to posterior. Slices were stuck on the slide using soaked filter paper in PBS (1X) or the slide is left on the bench for 1 day until it dries. The prepared slides were then placed in distilled water (dH_2_O) for 3 min for hydration then transferred to hematoxylin for up to 1 min. The slides were then placed in running tap water to provide the necessary alkalinity for “bluing” process. The slides were then immersed in eosin for up to 1 min. Slides were then placed in 95% ethanol for 2 times, 3 min each and then immersed in 100% ethanol for 5 min to prevent cell lysis. Finally, slides were placed in Xylol for 1 min, which acts as a clearing solvent to remove alcohol from tissues. Slides were then mounted and visualized on the microscope.

### Nissl stain

Brain tissue slides were first prepared, placed in distilled water (dH_2_O) for 3 min and then transferred to Cresyl violet (0.5%) for up to 2 min. The slides were then placed in running tap water and then immersed in distilled water. Slides were then placed in 75% ethanol for 1min, 95% ethanol for 2min, and then immersed in 100% ethanol for 3 min. Finally, slides were placed in xylol for 2 min and were mounted and visualized on the microscope.

### Data acquisition and quantification

For each mouse, images were acquired with a LSM710 confocal microscope, Carl Zeiss, Germany. At least four sections corresponding to the areas of interest were used for quantifications using Image J program (NIH). For Iba-1 and GFAP expression, the total area covered by these markers at the lesioned site was quantified with respect to the total injury area. For DCX marker, the area covered by the DCX+ cells throughout the lateral SVZ was quantified with respect to the total length of the SVZ. For NeuN quantification, the total NeuN+ area in the hippocampus was quantified on both contralateral and ipsilateral sides among different groups.

### Statistical analysis

All results are expressed as mean ± SEM. For data concerning IF, within each experimental group, statistical significance was evaluated using two-way analysis of variance (ANOVA) having the group (control, transplantation) as a parameter. The *p*-value was determined and values for *p* < 0.05, *p* < 0.001, *p* < 0.0001 (^*^, ^**^, ^***^ respectively within the same group and #, ##, ### respectively among groups) were considered significant.

## Results

### Establishment and validation of mild CCI model, neurosphere, and neuroblast assays

**Figure 2:**
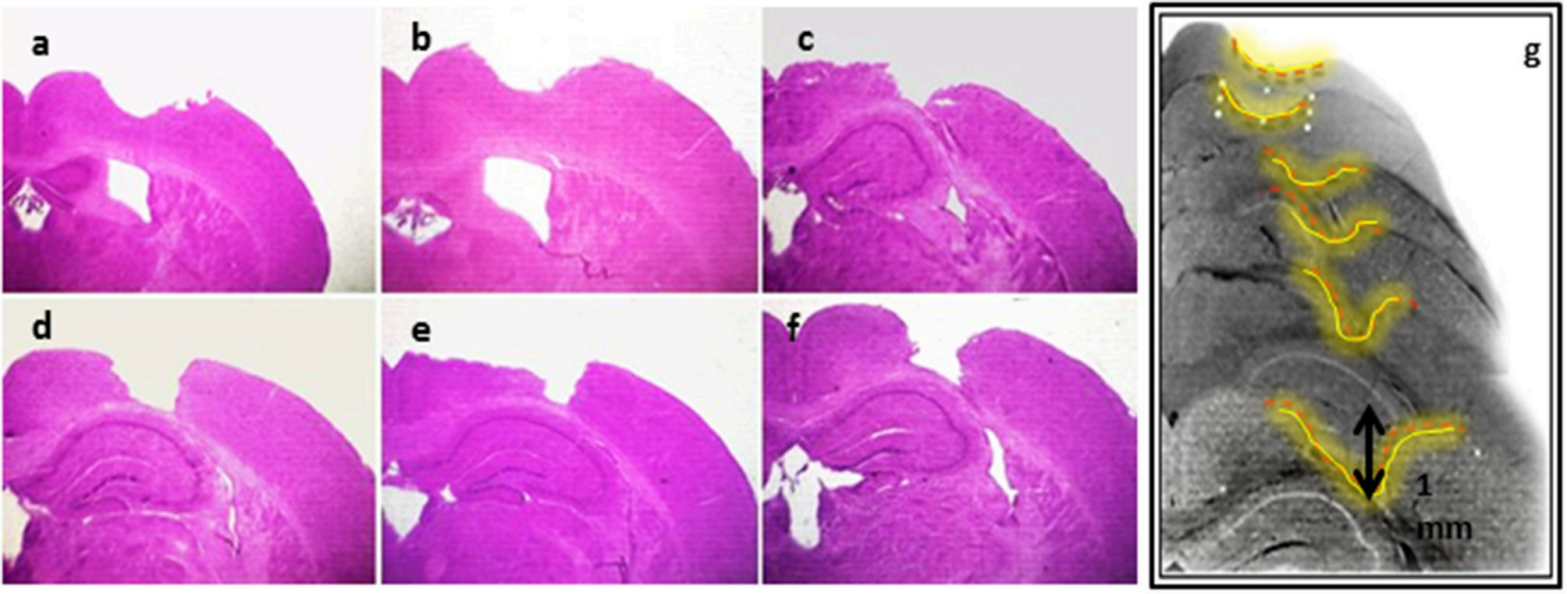
Establishment of CCI model. H&E staining showing serial sections **(a–g)** of the lesioned area of the brain tissues subjected to CCI, and detecting the morphological changes in the mouse brain coronal sections.

CCI lesion was established as described in the materials and methods section. CCI was then validated using H&E staining on histological sections of the mouse brain (Figure 2). Indeed, the expected morphological changes such as damaged parenchymal tissues and reduction in the cytoplasm of the injured cortex were observed in the lesioned somatosensory region (Figure 2). On the other hand, no significant malformations in the hippocampal region were observed. Moreover, Nissl stain confirmed that neurons residing in the ipsilateral cortex were highly injured (Supplementary Figure 1).

**Figure 3:**
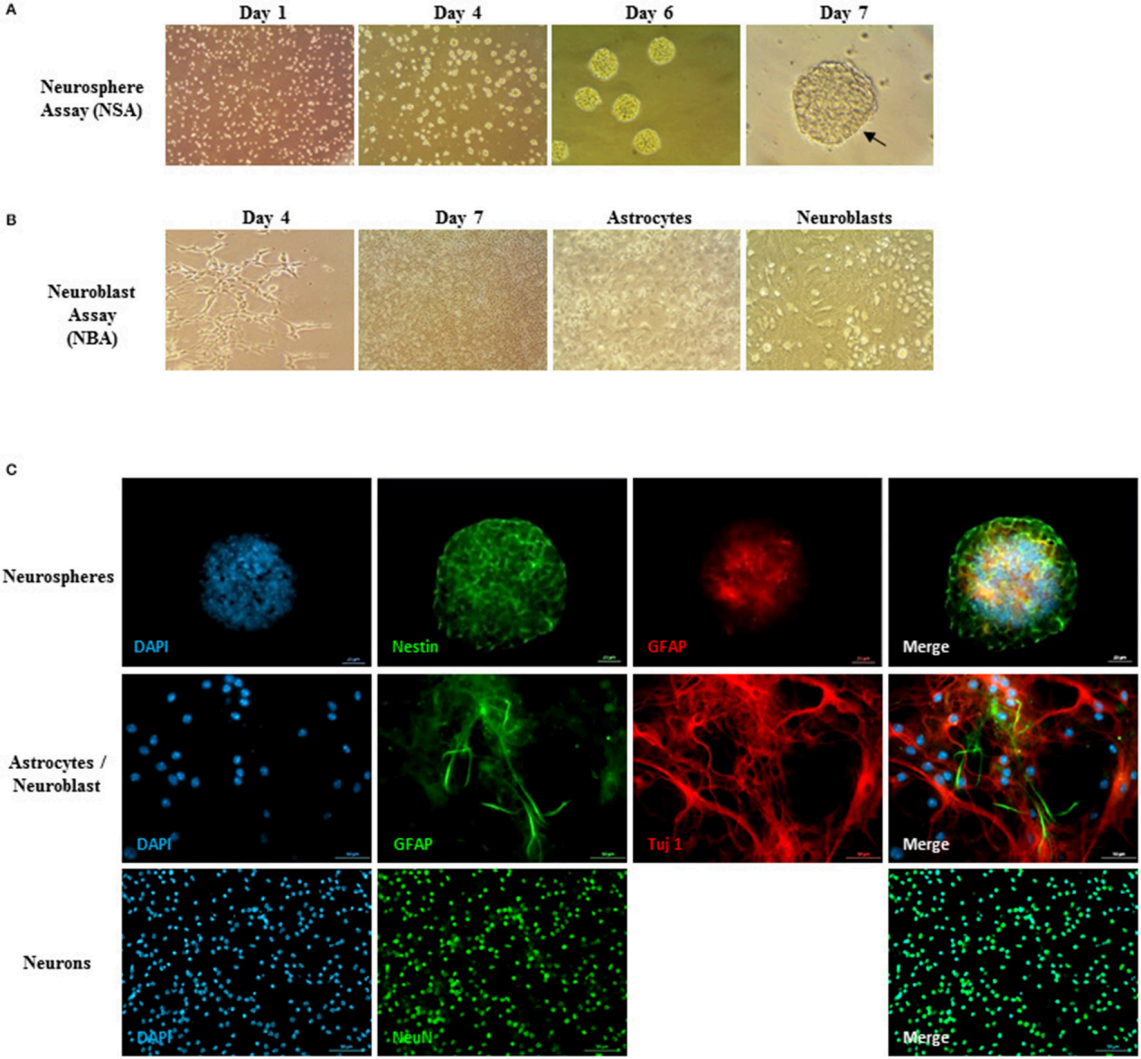
Neurosphere assay, neuroblast assay, and neurosphere immunostaining. **(A)** Young healthy neurospheres showing microspikes at 5–6 days post-culturing. **(B)** Neuroblast assay (NBA) with proliferation and differentiation stages. Flat astrocytic cells are shown by black arrows underneath, and round neuronal progenitor cells shown by red arrows on top. **(C)** Neurosphere immunostaining assay to validate the neural embryonic origin of the spheres. Upper panel: Neurospheres showed a Nestin, GFAP positive phenotype, thus reflecting the undifferentiated state of the ENSCs and showing that not all GFAP^+^-cells are differentiated astrocytes, but have a large majority of undifferentiated NSCs. Middle and lower panels: validation of the differentiated cells (DCs) phenotype residing in the NBA cultured population. The cells show a Tuj-1, GFAP positive phenotype (middle panel) as well as NeuN, GFAP positive immunostaining (lower panel).

In parallel, ENSCs were harvested from E14 embryos and cultured using the neurosphere assay. They showed microspikes morphology, characteristic of neurospheres, at 5–6 days of culture (Figure 3A). On the other hand, the three types of differentiated cells (DCs): flat astrocytic cells, round neuronal progenitor cells, and mature neurons were obtained using the neuroblast assay (NBA), after proliferation and differentiation stages (Figure 3B). The phenotype of cells was validated using immunocytochemistry where neurospheres showed nestin and GFAP staining, thus reflecting the undifferentiated state of stem cells whereas differentiated cells were positive for Tuj-1, GFAP, and NeuN markers (Figure 3C).

### Microglia is activated following CCI, but not enhanced after injection of ENSCs or DCs

**Figure 4:**
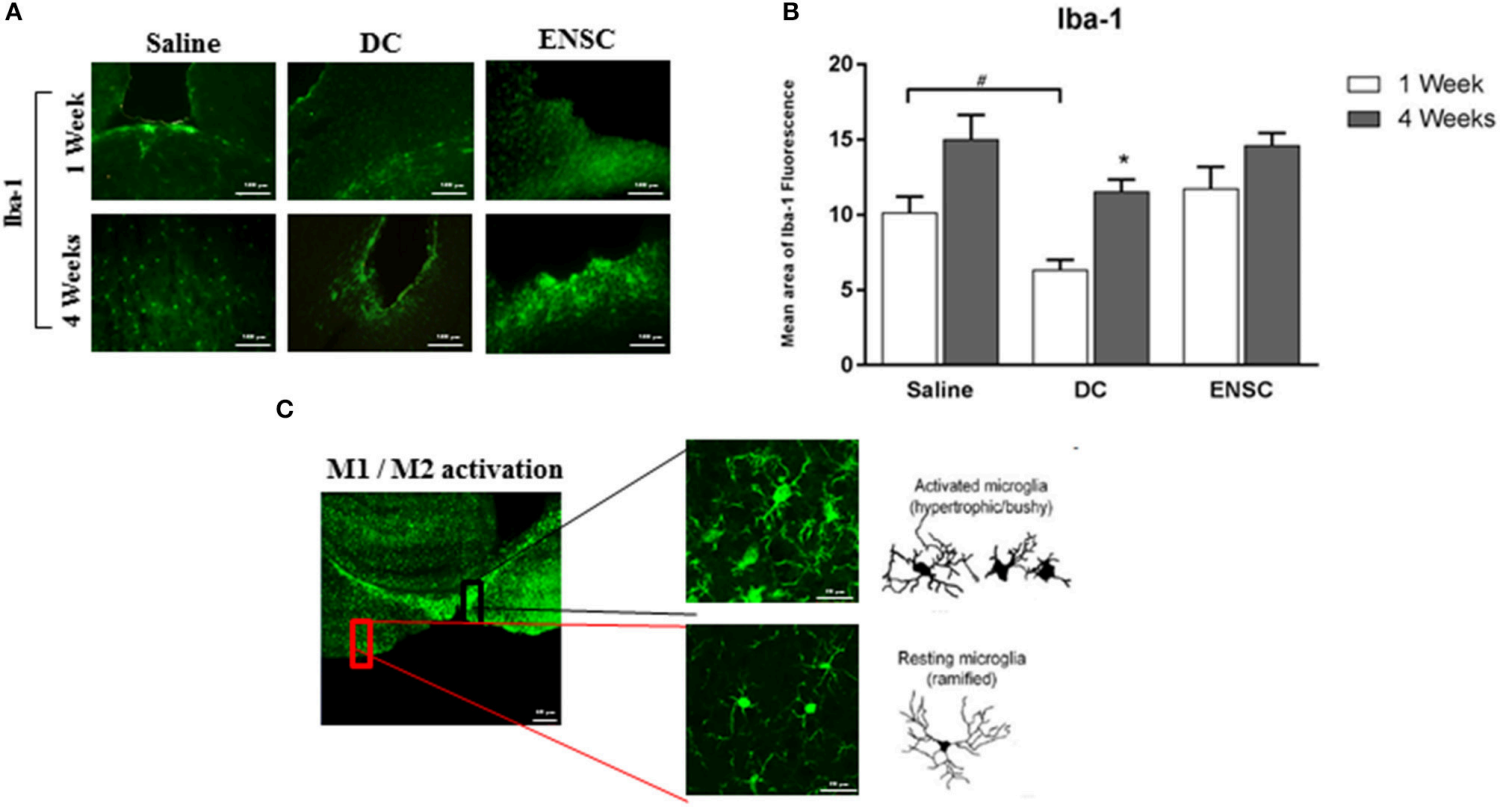
Expression and activation of Microglia (Iba-1) following CCI. **(A)** Microscopic images for Iba-1 immunostaining in the cortical injury site, at 1 and 4 weeks post-injection (40X). Scale bar is 100 μm. **(B)** Quantification of Iba-1 expression, at 1 and 4 weeks post-injection. Each bar represents the area of Iba1-positive cells per area of TBI whereas the Y-axis represents the total area covered by Iba1 marker at the TBI site with respect to the area of injury. Data are expressed as means ± SEM, *n* = 4 per group. *p < 0.05, ^#^*p* < 0.05. **(C)** Highly activated microglia in the injured cortex showing ramified, hypertrophic and bushy phenotype. Reduced resting ramified microglia were residing in distant areas from the lesioned region. Scale bar is 20 μm.

Iba-1 protein, a microglial specific marker, is usually used as a measure of neuroinflammation and neuroprotection during different phases of CCI. Microglial activation using Iba-1 was assessed by IF on the 6 groups of CCI mice (*n* = 4 each) injected with either saline, ENSCs or DCs and maintained for 1- or 4-weeks after injection (Figure 4A and Supplementary Figure 2). Quantification of IF data demonstrated a significant increase (^*^p < 0.05) in Iba-1 expression post-CCI in saline and DCs groups, but not in ENSC group, at 4 weeks in comparison to 1 week (Figure 4B). In addition, similar morphological changes were observed in different areas of the injured cortical region among the 6 groups of mice. Indeed, bushy hypertrophied Iba-1+ microglia were found to be highly expressed around the CCI region whereas resting microglia of amoeboid morphology were observed at distant places from the CCI zone (Figure 4C). Taken together, ipsilateral lesioned cortex expresses high levels of Iba-1+ microglia after 1 and 4 weeks of injection within the different groups (Saline, ENSCs, or DCs).

### Astrogliosis is significantly reduced by treatment with ENSCs at 1 week or DCs at 4 weeks post-transplantation

**Figure 5:**
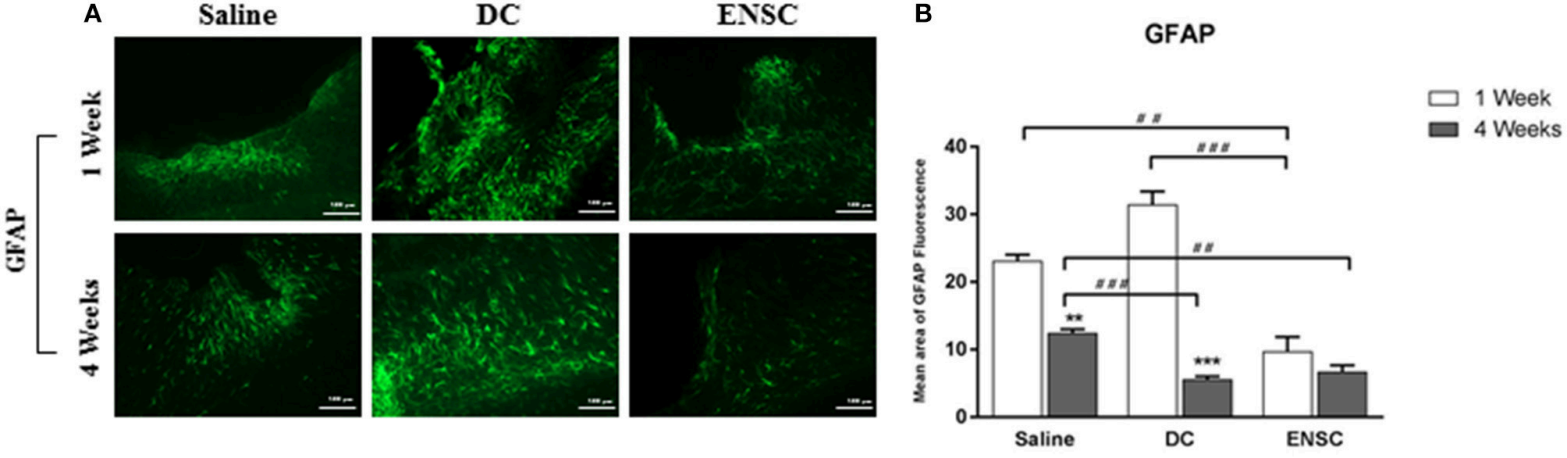
Expression of activated astrocytes (GFAP). Astrogliosis is significantly reduced by treatment with ENSCs or DCs, following mild CCI. **(A)** Microscopic images for GFAP immunostaining in the cortical injury site, at 1 and 4 weeks post-injection (40X). Scale bar is 100 μm. **(B)** Quantification of GFAP expression, at 1 and 4 weeks post-injection. Data are expressed as means ± SEM, *n* = 4 per group. **p < 0.01, ***p < 0.001, ^*##*^*p* < 0.01, ^*###*^*p* < 0.001.

Previous studies have shown that acute CCI induces inflammation, glial cell reactivity and a dramatic increase in the number of activated astrocytes, with hypertrophic cellar processes, leading eventually to neuronal apoptosis and “reactive astrogliosis” (45). The latter is characterized by GFAP protein, upregulated in the activated astrocytes, and occurs mostly in the ipsilateral cortex of the injured brain, as demonstrated by immunofluorescence (Figure 5A). When compared to the saline group, our data demonstrated a significant reduction in the number of GFAP expressing astrocytes in the ENSCs group, at 1 and 4 weeks post-injection (^**^p < 0.01, Figure 5B). This reduction was also highly significant in the ENSCs group at 1 week, in comparison to DCs (^***^p < 0.001). In addition, the DCs group also showed a highly significant reduction in GFAP expression at 4 weeks, in comparison to controls (^***^p < 0.001, Figure 5B). Taken together, treatment with ENSCs or DCs (glial and neuronal cells) following mild CCI demonstrated a reduction in astrogliosis, especially 4 weeks post-injection, as shown by a decrease in GFAP expression. This decrease in GFAP levels started very early in the ENSCs group (1 week), in comparison to DCs group, which was comparable in both groups at 4 weeks.

### Neurogenesis is significantly enhanced at the progenitor level in ENSCs before DCs groups

**Figure 6:**
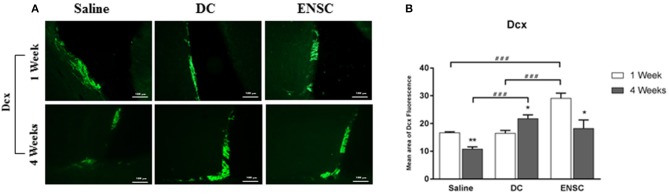
Expression of neuronal progenitors (DCX). Neurogenesis is significantly enhanced in ENSCs and DCs groups. **(A)** Microscopic images for DCX immunostaining in the SVZ region, at 1 and 4 weeks post-injection (40X). Scale bar is 100 μm. **(B)** Quantification of DCX expression in the SVZ, at 1 and 4 weeks post-injection. Data are expressed as means ± SEM, *n* = 4 per group. *p < 0.05, **p < 0.01, ^*###*^*p* < 0.001.

Previous studies reported an activation of neurogenesis and cellular proliferation in the subventricular zone (SVZ) and dentate gyrus (DG), following CCI (46, 47). Protein expression of DCX, a marker of late neuronal progenitors, was used to demonstrate the effect of injecting ENSCs or DCs, following CCI, on neurogenesis in the SVZ region (Figure 6A and Supplementary Figure 3). Our data demonstrated a significant increase in the number of DCX expressing progenitor cells at 1 week in the ENSCs group, in comparison to control or DCs groups (^***^p < 0.001) (Figure 6B). In addition, a significant increase in DCX was also obtained in DCs and ENSCs groups at 4 weeks post-injection, in comparison to controls (^***^p < 0.001 and ^*^p < 0.05; respectively) (Figure 6B). In summary, treatment with ENSCs or DCs after CCI increased the expression of DCX, therefore enhancing cellular proliferation, progenitor cell survival and neurogenesis at 1 and 4 weeks post-injection. Unlike DCs, ENSC showed an enhancement in neurogenesis 1 week after transplantation as well as reduced astrogliosis. In contrast, DCs treatment is less effective at this time point.

### Expression of mature neurons

**Figure 7:**
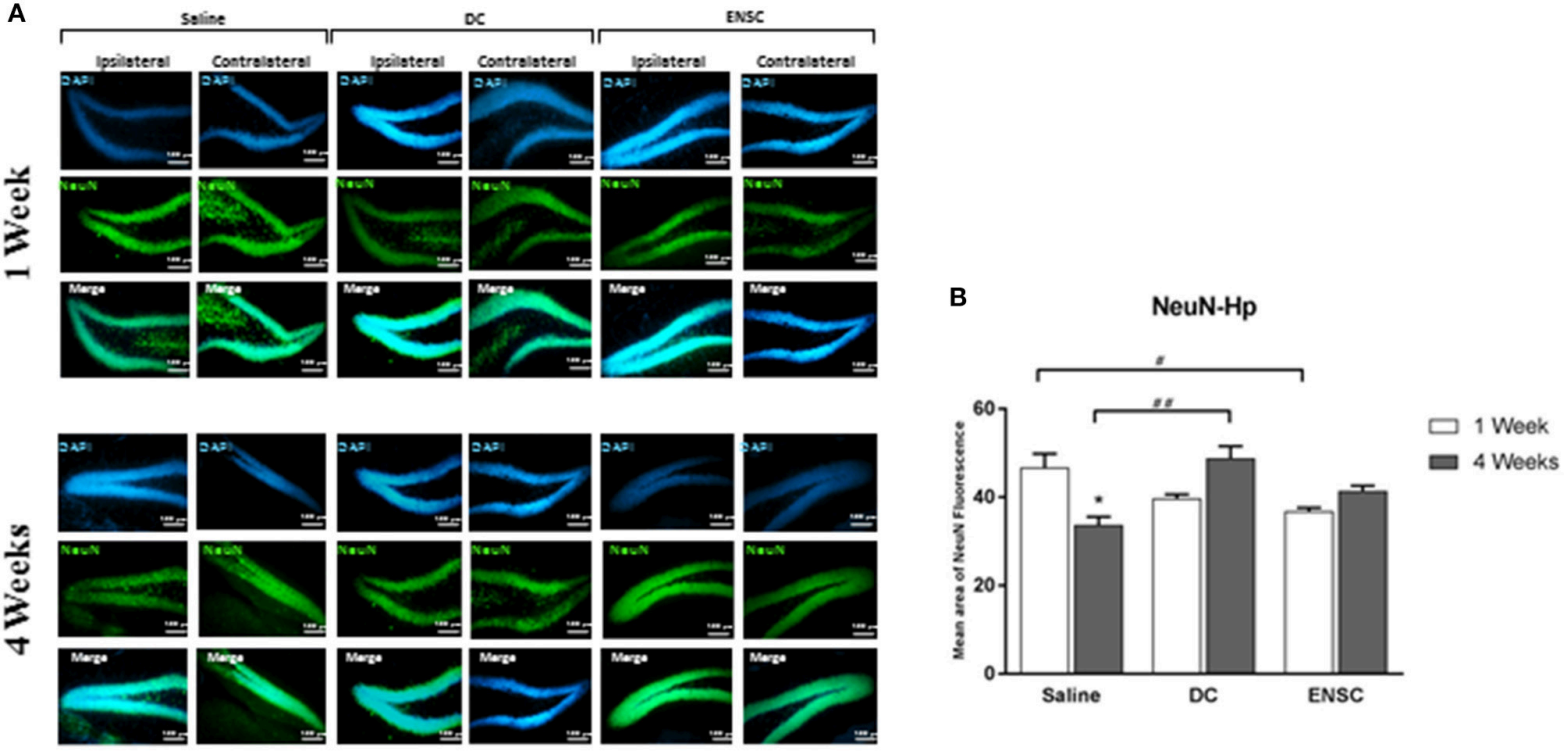
Expression of mature neurons (NeuN). **(A)** Microscopic images for NeuN immunostaining in the hippocampus (Dentate Gyrus), at 1 and 4 weeks post-injection (20X). Scale bar is 100 μm. **(B)** Quantification of NeuN expression in the hippocampus, at 1 and 4 weeks post-injection. Data are expressed as means ± SEM, *n* = 4 per group. *p < 0.05, ^#^*p* < 0.05, ^*##*^*p* < 0.01.

Protein expression of NeuN, a marker of mature neurons, was used to demonstrate the effect of injecting ENSCs or DCs, following CCI, on neuronal maturation in the hippocampus region (Figure 7A). Results showed a significant increase in the number of neuronal cells in DCs group at 4 weeks post-injection, in comparison to controls (^**^p < 0.01, Figure 7B). On the other hand, our data demonstrated a significant decrease in NeuN expressing cells in control mice at 4 weeks post-saline injection, in comparison to 1 week, but also in ENSC group at 1 week, in comparison to controls (^*^p < 0.05, Figure 7B). Finally, no significant increase in NeuN levels was observed in the other groups and time points (Supplementary Figure 4). Taken together, injection of ENSCs or DCs post-CCI suggests that it may increase neurogenesis via inducing neural progenitor's proliferation and expression rather than neuronal maturation.

## Discussion

Traumatic brain injury, an intracranial injury, has been recognized among the major causes of death worldwide with broad devastating symptoms and disabilities. TBI causes primary injury due to mechanical forces at the time of the incident followed by a secondary injury. The latter is characterized by cytotoxicity, cerebral edema, oxidative stress, necrosis, apoptosis, mitochondrial dysfunction, and inflammation (9–11). These neuropathological sequelae happen progressively, aggravating the neural injury and inducing neuropsychological and motor deficits. Thus, several options have been proposed as a prospective therapy for TBI including stem cell transplantation using mesenchymal stem cells (MSCs) (26, 48), neural stem cells (NSCs) (27–30), induced pluripotent stem cells (iPSCs) (31) and embryonic neural stem cells (ENSCs) (32). Thus, the aim of our study was to investigate the effect of grafting different cell types (ENSCs or DCs) on neuroinflammation and neurogenesis after mild CCI. This study describes the environment surrounding the injury after transplanting ENSCs or DCs in the same conditions.

Neuroinflammation is considered as a potential therapeutic target due to its correlation with neurological symptoms and pathology (49). In fact, inflammatory response post-TBI is signaled by glial cell activation, peripheral leukocyte recruitment via disturbed BBB and rapid rise in the levels of inflammatory mediators such as cytokines and chemokines (50, 51). Mediators and cellular events activate the immune cells and induce their migration toward the injured area (52). For instance, neutrophils are the first cells infiltrating the injured brain followed by migration of microglia and activation of astrocytes (52). Neuroinflammation can be either harmful by inducing oxidative damage and neuronal death or beneficial by promoting the clearance of debris and tissue remodeling (53).

Our data showed an up-regulation of microglial activation which is in accordance with a recent study in moderate TBI, using the CCI model, demonstrating that microglial activation occurred 1-week post-CCI, which significantly increased 5 weeks after injury and persisted for 1 year (54). Since there is an interaction between astrocytes and microglia post-CCI, DCs which contain populations of astrocytes, but not ENSCs, affect the microglial expression more significantly at 1 week. It was previously demonstrated within the cortex of a moderate diffuse TBI mouse model that the expression of tumor necrosis factor alpha (TNFα) and IL-6 peaked at 3–9 h post-TBI (55). Similarly, increased microglial activation was shown at 6 h post-TBI in pigs, indicating an association between the induction of an inflammatory response and brain pathology (56). Microglial activation is well established as one of the secondary biochemical changes following TBI, providing an evidence of neuroinflammation post-TBI (54). Indeed, microglial activation is characterized by morphological as well as functional modifications where the resting ramified microglia transforms into hypertrophic bushy cells involved in secretion of pro- and anti-inflammatory molecules (54). In fact, M1-like microglial activation is implicated in the deterioration of injury due to the release of cytotoxic mediators (57, 58) whereas M2-like microglial activation contributes to injury recovery and repair due to the release of neurotrophic and immunomodulatory factors (59, 60). Several TBI studies reported a peak in M2 microglia during the first 7 days after injury; however, this phenotype shifts to M1 microglia thereafter (61, 62). In another model of stroke, the M2 marker CD206 was highly expressed 3 days after ischemia whereas the M1 marker MHCII was found highly expressed at day 7 post-ischemia (63). Our observation of microglial up-regulation following mild CCI suggests that induced neuroinflammation may last for a long time after the initial brain insult, increasing the risk of persisting neurodegeneration and therefore the need of targeting inflammatory events using new therapies. Chronic microglial activation is associated with up-regulation of pro-inflammatory cytokines such as IL-1β and TNFα (64) contributing to long-term neurodegeneration following TBI.

On the other hand, our data demonstrated a significant reduction in the number of GFAP expressing astrocytes in control and DC groups at 4 weeks post-injection, in comparison to 1 week. In addition, GFAP decreased in the ENSC group at 1 week, in comparison to control or DC groups, which was maintained at 4 weeks. Previous studies have shown that TBI causes a dramatic increase in the number of activated astrocytes with hypertrophic cellar processes resulting in “reactive astrogliosis” (45). In fact, astrocytes affect many essential neural functions in normal CNS by maintaining the extracellular balance of ions and neurotransmitters, regulating the blood flow and influencing synaptic activity and plasticity (65). Therefore, dysfunction in the mechanisms underlying scar formation and reactive astrogliosis cause detrimental effects on the CNS (65). During the acute phase of TBI, inflammation and glial cell reactivity are induced due to the alteration in the BBB leading eventually to neuronal apoptosis (45). Our results demonstrated that following mild CCI, treatment with ENSCs or DCs (glial and neuronal cells) caused a reduction in astrogliosis, suggesting that our cell therapy at 1 and 4 weeks' time intervals provide a neuroprotective (anti-inflammatory) role which helps in recovery post-CCI.

One of the major results of this study was that Doublecortin (DCX) expression increased significantly in the CCI groups after transplantation. In fact, our data showed a significant up-regulation in the number of DCX expressing progenitor cells in DCs and ENSCs groups at 4 weeks post-injection, in comparison to controls, in addition to a significant increase at 1 week in the ENSCs group, in comparison to control and DC groups. Indeed, it is well known that the subgranular zone (SGZ) of the DG in the hippocampus, in addition to the SVZ region, is a site of neurogenesis in adults where there is a continuous regeneration of neuronal cells (66). Recent evidence showed that neurogenesis occurs near the damaged brain regions after TBI in humans, where the newly produced cells differentiate into mature neurons after migrating into the boundary zone of the lesioned cerebral cortex (67). In addition, spontaneous behavioral recovery is initiated after activation of endogenous neurogenesis following TBI (68). Despite this initial cellular proliferation and neurogenesis after trauma, survival and maturation of cells are not maintained since this early endogenous recovery is not sufficient to sustain the progenitor cell population (69). It was shown that only 20% of the newly born striatal neurons survived for 2 weeks following middle cerebral artery occlusion in rats (70). Similarly, after intracerebral hemorrhage, most newly generated cells, observed between 72 h and 1 week, did not survive more than 3 weeks (71). It's important to note that it takes 4 weeks for the new progenitors to become functionally mature neurons (72). Moreover, the generated immature neurons are sensitive to environmental factors such as the release of inflammatory mediators by activated microglia and astrocytes which affect the newly produced progenitor cells, suppressing long-term neurogenesis after TBI (69). Interestingly, targeting the inflammatory events after brain injury may augment neurogenesis and maintain its activation. Several studies have demonstrated the complex role of microglia in induced neurogenesis following TBI (20).

Using NeuN as a marker of mature neurons, we validated the effect of cell transplantation on neurogenesis. Although it takes 4 weeks to obtain mature and functional neurons, the control group showed a decrease in NeuN expression after 4 weeks of injury. On the other hand, the groups receiving DCs demonstrated a significant increase in NeuN expression.

One limitation in our study, however, was the incapacity to distinguish engrafted neurons and their axons from host tissue. In fact, it was not possible to characterize the transplanted neurons, to study their survival and migration and to assess possible developed projections of the engrafted cells. Alternatives to overcome this limitation would be to use GFP mice (73) or fluorescently labeled cells for transplantation (74). As for the survival of the engrafted cells, it is affected by the reduced perfusion, i.e., low vascularization and oxygen levels, and the persisting neuroinflammation in the lesion core. The inflammatory response seems to impact neuronal survival depending on a balance between pro- and anti-inflammatory mechanisms induced by different factors. Chronic neuroinflammation induced after injury increases the risk of persisting neurodegeneration and therefore reduces the survival of engrafted cells. Meanwhile, in another model of lesion, it was shown that a 1-week delay between the cortical lesion and embryonic neurons transplantation can significantly enhance graft vascularization, cell proliferation, survival and density of projections developed by grafted neurons, leading to a beneficial impact on functional repair and recovery (75).

Taken altogether, 4 weeks after injection of ENSC or DCs, our data revealed no preference for the transplantation of one cell type over the other. In fact, microglial activation was enhanced in both groups whereas astrogliosis was reduced, which also suggests that ENSCs and DCs may play a critical role in targeting the inflammatory events and in enhancing early progenitor cell survival. Moreover, transplantation of both cell types, ENSCs or DCs, enhanced neurogenesis. Hence, to repair the damaged brain, concomitant use of both ENSC and DCs, targeting post-injury neuroinflammation and enhancing the survival of newly generated cells by supporting an optimal environment for the cells, may provide a new perspective for cell therapy.

## Author contributions

MN, SM, FB, FN, NR, KZ, and FK carried out the experiments. MN, NB, KZ, and JS analyzed data. KZ and FK designed and supervised the study. FK, MN, and KZ wrote the manuscript. The work was conducted in the laboratory of FK at the American University of Beirut. KZ and FK have contributed equally to this work.

### Conflict of interest statement

The authors declare that the research was conducted in the absence of any commercial or financial relationships that could be construed as a potential conflict of interest.

## Supplementary material

Supplementary Figure 1 Nissl staining. Brain tissue sections of CCI mice injected with saline, DCs or ENSC, respectively, 1 week post-CCI. Cortex at CCI region (ipsilateral) with 10× magnification showing the injured area depth of 1mm. Higher magnification confirmed damaged parenchymal tissues in the cortex post-CCI (data not shown).

Supplementary Figure 2 Expression of Microglia (Iba-1) in the cortex following transplantation. **(A,B)** Microscopic images for Iba-1 immunostaining, at 1 and 4 weeks post-injection. Scale bar is 100 μm. **(C)** High magnification images from regions of interest showing immunolabeled microglia. Scale bar is 20 μm.

Supplementary Figure 3 Expression of neuronal progenitors (DCX) following injection. Microscopic images for DCX immunostaining, in **(A)** saline, **(B)** DCs, and **(C)** ENSCs groups, at 1-week post-injection. Scale bar is 100 μm.

Supplementary Figure 4 Expression of mature neurons. **(A)** Microscopic images for NeuN immunostaining, at 1 and 4 weeks post-injection. Scale bar is 100 μm. **(B)** Quantification of NeuN expression in the Cortex, at 1 and 4 weeks post-injection. Data are expressed as means ± SEM, *n* = 4 per group. ^*^*p* < 0.05, ^**^*p* < 0.01, ^***^*p* < 0.001.

## Abbreviations

ENSC: embryonic neural stem cells
DC: differentiated cells
BBB: blood-brain barrier
CCI: controlled cortical impact
IF: immunofluorescence
GE: Ganglionic eminences
GFAP: glial fibrillary acidic protein
DCX: doublecortin
NeuN: neuronal nuclei
PBS: Phosphate-buffered saline
Iba-1: ionized Ca binding adaptor molecule
H&E: Hematoxylin and Eosin
SVZ: subventricular zone
DG: dentate gyrus.
