# Supplementary figures and images for: Transplantation of Embryonic Neural Stem Cells and Differentiated Cells in a Controlled Cortical Impact (CCI) Model of Adult Mouse Somatosensory Cortex
Source: Front Neurol. 2018 Oct 24;9:895. doi: 10.3389/fneur.2018.00895 (PMC6208009; doi:10.3389/fneur.2018.00895)

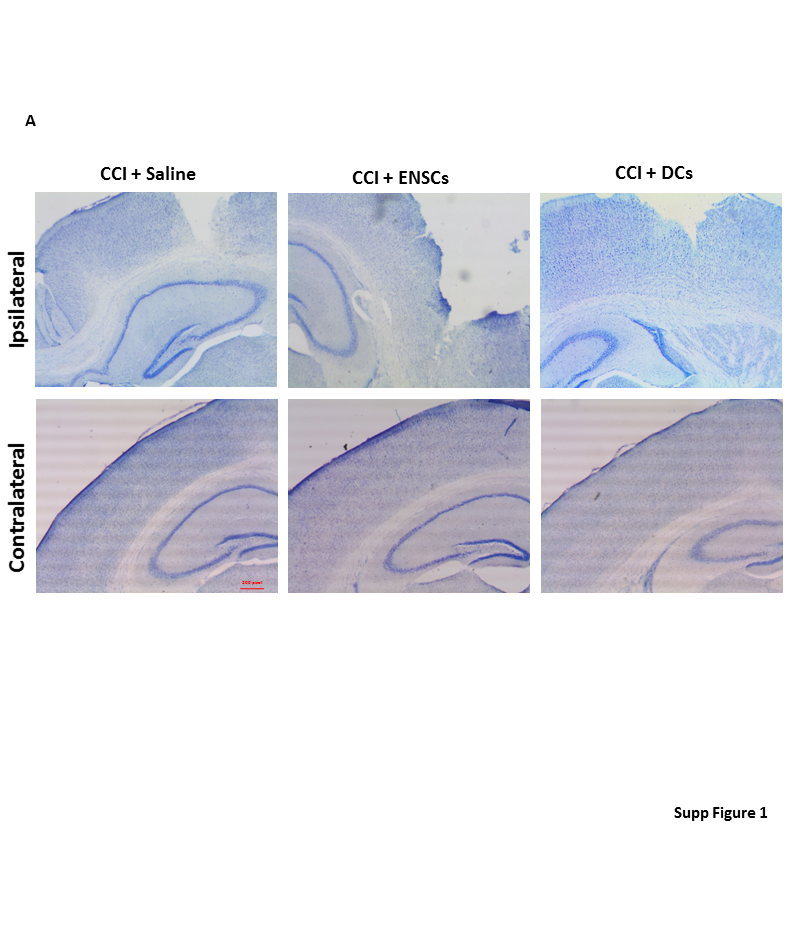

Supplement: Supplementary Figure 1 — Nissl staining. Brain tissue sections of CCI mice injected with saline, DCs or ENSC, respectively, 1 week post-CCI. Cortex at CCI region (ipsilateral) with 10× magnification showing the injured area depth of 1mm. Higher magnification confirmed damaged parenchymal tissues in the cortex post-CCI (data not shown). [file Image_1.TIF]

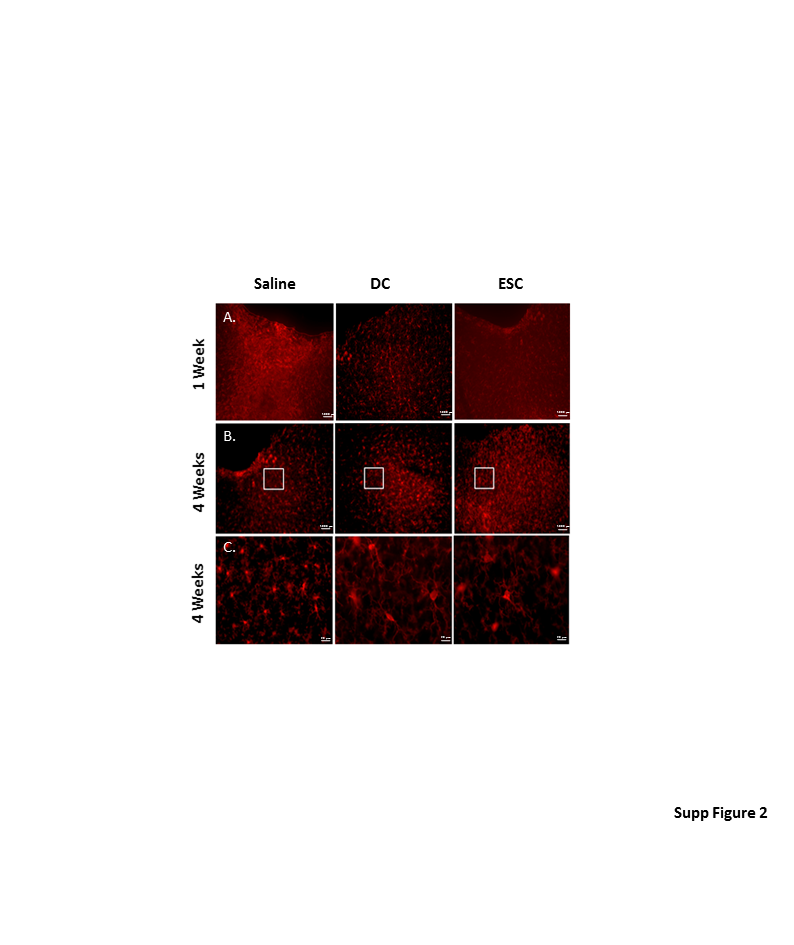

Supplement: Supplementary Figure 2 — Expression of Microglia (Iba-1) in the cortex following transplantation. (A,B) Microscopic images for Iba-1 immunostaining, at 1 and 4 weeks post-injection. Scale bar is 100 μm. (C) High magnification images from regions of interest showing immunolabeled microglia. Scale bar is 20 μm. [file Image_2.TIF]

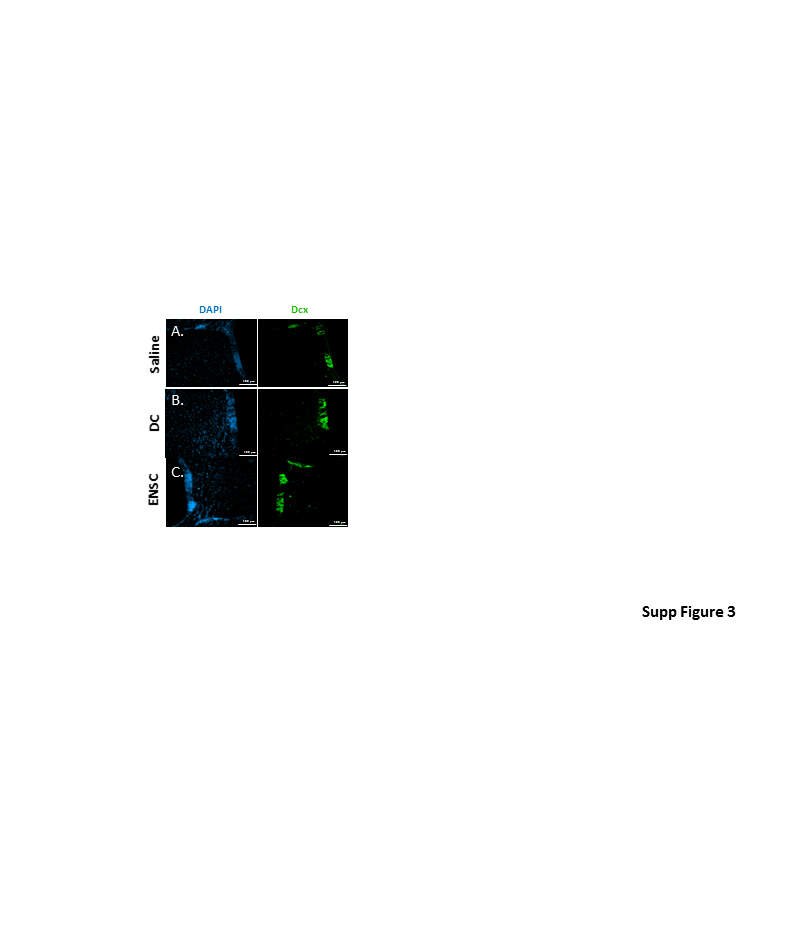

Supplement: Supplementary Figure 3 — Expression of neuronal progenitors (DCX) following injection. Microscopic images for DCX immunostaining, in (A) saline, (B) DCs, and (C) ENSCs groups, at 1-week post-injection. Scale bar is 100 μm. [file Image_3.TIF]

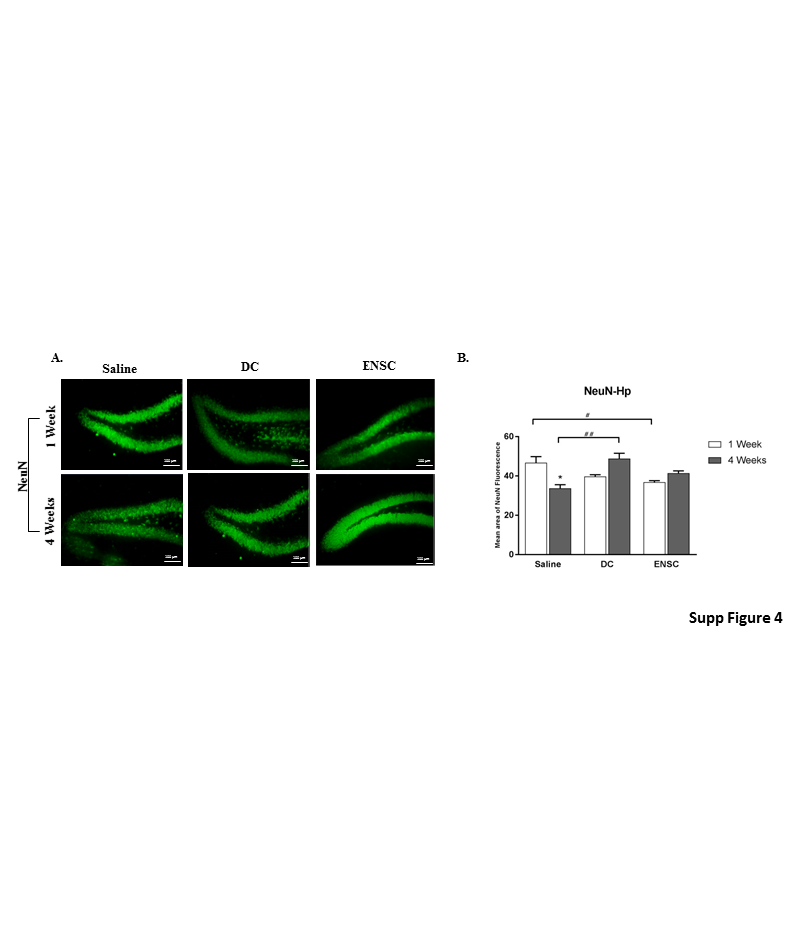

Supplement: Supplementary Figure 4 — Expression of mature neurons. (A) Microscopic images for NeuN immunostaining, at 1 and 4 weeks post-injection. Scale bar is 100 μm. (B) Quantification of NeuN expression in the Cortex, at 1 and 4 weeks post-injection. Data are expressed as means ± SEM, n = 4 per group. *p < 0.05, **p < 0.01, ***p < 0.001. [file Image_4.TIF]
